# Stigma-Stop: A Serious Game against the Stigma toward Mental Health in Educational Settings

**DOI:** 10.3389/fpsyg.2017.01385

**Published:** 2017-08-21

**Authors:** Adolfo J. Cangas, Noelia Navarro, José M. A. Parra, Juan J. Ojeda, Diego Cangas, Jose A. Piedra, Jose Gallego

**Affiliations:** ^1^Department of Psychology, University of Almería Almería, Spain; ^2^Department of Education, University of Almería Almería, Spain; ^3^Department of Informatics, University of Almería Almería, Spain

**Keywords:** stigma, virtual reality, psychological disorders, serious games, human factors

## Abstract

This paper presents the results from the application of a serious game called Stigma-Stop among a group of high school students with the aim of reducing the stigma toward mental illnesses. The video game features characters with various mental disorders (schizophrenia, depression, bipolar disorder, and panic disorder with agoraphobia) and provides information about these problems. Additionally, the game asks players about whether they have ever felt the same as the characters, if they believe the characters are psychologically well, and if they think they could help these individuals. Similarly, a variety of reactions are provided for players to choose from when they encounter the characters with these problems. A total of 552 students between the ages of 14 and 18 participated in the study, and they were randomly assigned to either the experimental group, which used Stigma-Stop, or the control group, which utilized a video game completely unrelated to mental health. Both video games were used for similar lengths of time. Following the application of Stigma-Stop, a statistically significant decrease was obtained in levels of stigma toward schizophrenia, both in terms of stereotypes and, to a greater extent, its potential dangerousness. However, this was not the case in the control group. Results thus demonstrate the video game’s usefulness toward eradicating erroneous notions about serious mental disorders like schizophrenia.

## Introduction

In today’s world, one of the most important problems related to mental health is the stigma that is placed on individuals who suffer from mental disorders, which complicates both treatment and rehabilitation. In this regard, some of the most widespread misconceptions are that these people are aggressive, strange, unpredictable, weak, lazy, unproductive, incurable, guilty of their illness, and are people who cannot be reasoned with (Crisp et al., 2000; Byrne, 2001; Vezzoli et al., 2001; López et al., 2008, 2012).

These misconceptions constitute one of the greatest obstacles for making progress in the recovery of these individuals, since, although effective psychological and psychiatric treatments do exist, social integration is complicated because of the social rejection toward people with mental disorders. This leads to terrible consequences when the time comes to find work or a home, or maintain relationships with either friends or a partner (Penn and Martin, 1998).

Furthermore, most mental disorders, particularly the most serious ones, such as schizophrenia, anorexia, and substance abuse, generally begin during adolescence, which is the critical period in our society when young people become future citizens. For this reason, most anti-stigma programs are applied among this population (Pinfold et al., 2003; Schulze et al., 2003; Watson et al., 2004; Spagnolo et al., 2008; Pinto-Foltz et al., 2011).

The numerous anti-stigma programs that currently exist utilize different strategies that seek to provide information about mental disorders (by means of informative pamphlets, comics, videos, public activities, etc.). It is also quite common for individuals with mental problems themselves to speak to young people about their difficulties, how they overcame them, and their general experience. This creates direct interaction between people afflicted by mental problems and individuals without these difficulties (Pettigrew and Tropp, 2006; López et al., 2008; Michaels et al., 2012).

However, video games have not yet been utilized to provide information and educate young people about these mental disorders. There *are* serious games for preventing and treating various psychological disorders, particularly depression and anxiety (Connolly et al., 2012; Fleming et al., 2014), but none exist which combine different psychiatric problems and also focus specifically on the issue of stigma.

This seems odd considering that the game medium is the most common form of entertainment among young people and that to which they dedicate the most time (Entertainment Software Association [ESA], 2015). In addition, Serious Games possess several advantages compared to other informative and/or preventive strategies: they provide immediate feedback; which further motivates the users they are aimed at; they improve the learning process; are easy to use; inexpensive once developed; and can reach a vast population (Carmona et al., 2010).

The available literature reveals the existence of a large number of recent studies that support effectiveness of video games in the evaluation and treatment of psychological disorders (Ceranoglu, 2010; Stallard et al., 2010; Geurts et al., 2011; Connolly et al., 2012; Fernández-Aranda et al., 2012; Sereni et al., 2014; Aiken and Berry, 2015; Choi and Lee, 2015; Karimpur and Hamburger, 2015). For example, virtual reality has been utilized in the treatment of various phobias such as acrophobia (Levy et al., 2016), social anxiety disorder (Gebara et al., 2016), agoraphobia (Quero et al., 2014), and arachnophobia (Miloff et al., 2016).

Apart from the treatment of different psychological problems, virtual reality has also been applied in learning environments (Morris et al., 2013; Ashinoff, 2014). For example, Jou and Wang (2013) conducted a program whose objective was to reinforce the learning of technical skills. They utilized virtual reality to verify how effective it was in improving five dimensions of learning: knowledge, understanding, simulation, application, and creativity. Forty students participated in the study which lasted a semester, and the program obtained positive results.

However, no games or programs have focused on providing players with information about mental disorders that seeks to create empathy among young people toward individuals who suffer from such problems. If this were done, an open dialog concerning these topics could be established by treating such disorders as something normal, which could ultimately dispel any possible misconceptions. In the case of the present study, the objective was to develop a serious game called Stigma-Stop, which would enable young people to become familiar with mental disorders by increasing their knowledge and comprehension. It was also crucial to emphasize the importance of biographical and/or contextual experiences related to these disorders, along with the idea that any person can suffer from similar experiences throughout their life. Furthermore, the game also had to demonstrate examples of how to react in different situations, promoting interest in actively helping individuals with certain disorders.

The video game presents four of the most common disorders among young people, two of which are considered common mental illnesses (i.e., depression and agoraphobia), while the other two are serious (i.e., schizophrenia and bipolar disorder). The latter two, apart from being highly prevalent among this age group, also tend to manifest themselves precisely during adolescence (National Institute for Health and Care Excellence [NICE], 2013). These reasons define the relevance of all four disorders and justify their inclusion in this study. Interstingly, although there is a great deal of concern regarding these illnesses in society, ironically, there is a considerable lack of information about the truth behind them (Spitzer and Cameron, 1995; Adler and Wahl, 1998).

### Participants

The sample was comprised of 552 students between the ages of 14 and 18 (*X* = 15.78; *SD* = 2.65) from 24 classes belonging to 12 secondary schools in the province of Almería (Spain). Of this group, 50.03% were female and 50.7% were male. The participants were randomly divided into an experimental group (484 students from 21 classes) and a control group (68 students from 3 classes from 3 different secondary schools). There were two reasons for making the experimental group much larger than the control group. Firstly, it was decided that it would be practical to reach as many students as possible so that they might benefit from the effects of the experience, insomuch as the program was something new and appealing. Secondly, it was expected that the responses of the control group would be highly stable, which was precisely the case as hardly any changes occurred among these participants.

### Instruments

#### Questionnaire on Student Attitudes toward Schizophrenia (Schulze et al., 2003)

This is a tool that was originally created in Germany for secondary school students as part of the Global Program of the World Psychiatry Association against stigma and discrimination. The present study utilized a Spanish version of the validation tool (validated by Navarro et al., 2017) which ultimately discovered two factors: the first being sterotypes and the other aggressiveness, with a general Cronbach’s Alpha of 0.95.

#### Stigma-Stop

**FIGURE 1:**
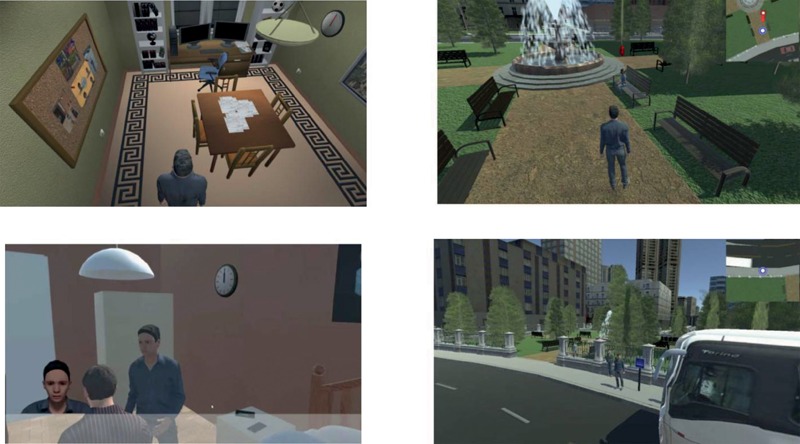
Stigma-Stop scenes.

Stigma-Stop is a Serious Game developed with Unity3D software for three platforms: PC, website, and Smartphone application. It presents four characters who suffer from different mental disorders, namely depression, schizophrenia, bipolar disorder and panic disorder with agoraphobia. The objective of the player is to convince the characters to work toward a common goal, which is to participate in a video game design contest. At the beginning of the game, the player can choose between either a male or female avatar, depending on their preferences, with the objective of increasing their involvement and making the experience more personal and realistic. **Figure 1** displays several frames from the game. There is a version to use in Spanish and another in English. Both will be available soon to download for free from the website http://stigmastop.net/.

### Development Environment

This video game was developed with the game engine Unity3D (Wang et al., 2010). This specific tool was chosen because it allows the product to be designed for more than 10 platforms, among which are Windows, Linux, Mac, Android, iOS, WebGL, and PS4. Furthermore, as one of the objectives was to publish this serious game for PCs, the Internet and mobiles, this game engine also suited the needs of the project. Apart from these characteristics, and in addition to being reasonably priced, Unity3D provided an environment with a rather fast learning curve during the initial phases and very high graphic quality, especially in terms of the changes made to the 3D rendering, and lights and camera in its latest version, Unity 5. The 3D models in the virtual world were designed using the tool Blender (Takala et al., 2013).

The serious game designed for the present study classifies as non-immersive virtual reality because it consists of a completely 3D environment in which the user does not need any type of special device to interact. This type of virtual environment was chosen primarily because the studies were going to be conducted with large groups of students and it was not possible to offer the necessary devices (VR headsets) to each of the subjects. This, in turn, made the experimentation process slower and more complicated.

### User Interaction

The user can interact with different environment elements such as the message board in the protagonist’s room, which allows them to call one of their friends, or the bus stop, to get the bus that takes them to see one their friends. The player can also interact with the other characters in the game through dialogs that allow them to choose among three options, although only one is correct. The user can play four different mini-games (which will be described later on) and complete surveys about the characters to assess the serious game itself.

A standard mouse and keyboard can be used to interact with these elements, but a gamepad can also be utilized as it makes moving the character through the different scenes easier. As for the mobile version, the touch screen was programmed as the application’s interaction medium.

### Flow Program

The player must visit each one of the four young people with different mental disorders in order to interact with the characters and indicate the reaction they consider most appropriate in the given situations. Each character can only be visited once and when the user meets one of them, an animated dialog begins during which some illness symptoms are shown at the beginning of the conversation and some at the end. The user can choose among three options to continue the action, but only one of the options is correct. If the player selects the incorrect option, an explanation is shown explaining why it is not the most suitable choice and it is recommended they choose another.

**FIGURE 2:**
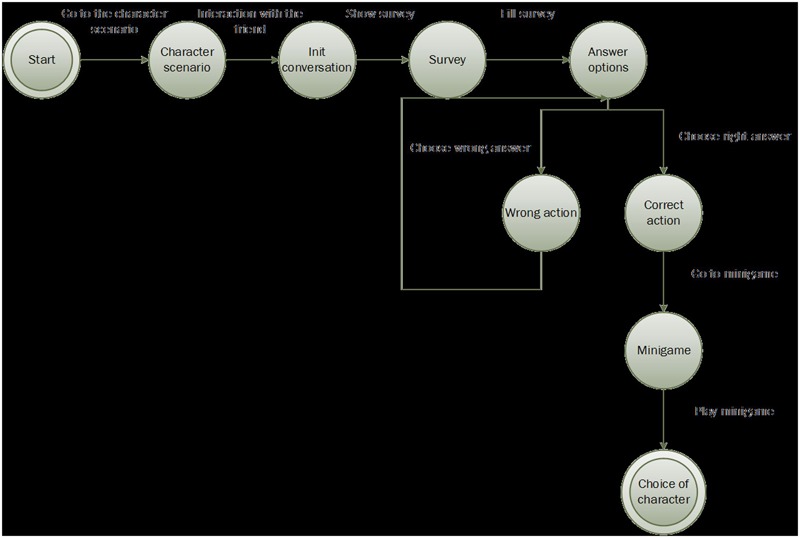
Logic flow for each character.

At the beginning of the game, the user has to first register to gain access. As previously mentioned, the objective of the protagonist is to visit four friends in order to design a video game. In this case, it was decided that the user would be given the freedom to choose the character they wanted rather than fixing a specific line of action that forced the user to follow a predefined sequence. To this end, a machine of finite states was utilized (Soon et al., 2013) in which each state represented a situation in the game that the user had to experience. The sequence is the following (see **Figure 2**):

1. Once the user chooses a character, they have to find that individual on the map, which is why the game includes a mini-map that constantly indicates the position of the character the player must reach.
2. Once the friend has been found, an animated dialog begins with that character in which symptoms of the illness are presented. When the dialog finishes, a short form appears with questions about general knowledge concerning each disorder and the user is also able access more information about the current condition of the characters and what circumstances led them to develop these types of problems.
3. After meeting each character, the player is asked if they think the character is emotionally wel; if they themselves have ever felt the same; if they think they would be able to help that individual (and specify how).
4. The user can choose among three options to continue the action, but only one of the options is correct. If the player selects the incorrect option, an explanation is shown explaining why it is not the most suitable choice and it is recommended that they choose another. When the player chooses the correct option, they are transferred to the “mini-game” state where they can play with one of the mini-games included in the program.
5. Once this phase is finished, the user chooses another character, bearing in mind that the previously selected one is deactivated at that point as they can visit each friend only once.

### Minigames

This serious game includes four minigames with a unique feature, namely, each of them makes reference to a mental illness and allows the user to learn information about each particular disorder, in addition to other details provided over the course of the game.

**FIGURE 3:**
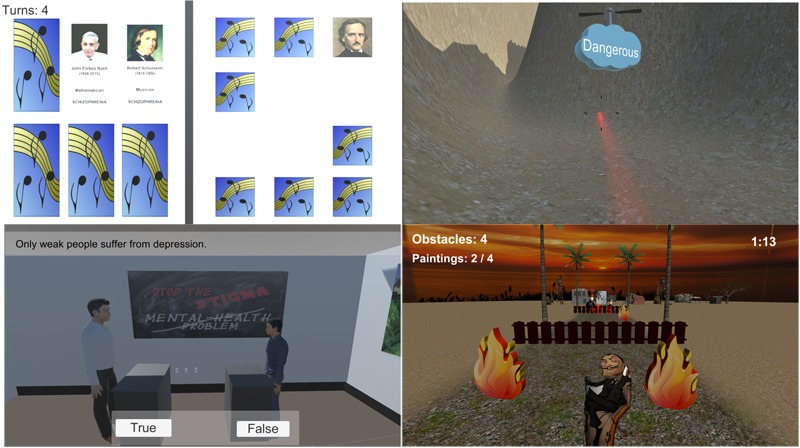
Stigma-Stop minigames.

The four minigames (see **Figure 3**) are:

*Memory*: This minigame follows the same procedure as memory games in which the player has to match two identical pictures to make a pair. In this case the characters that appear on the cards are famous people, such as Edgar Allan Poe, Vincent Van Gogh, Leonardo Da Vinci, etc., that have some type of mental illness. When both cards of the same pair are matched, a car for that character is shown containing personal information (name, profession, mental illness, etc.).

*Trivia*: In this minigame the user has to answer a series of questions related to certain aspects about mental illnesses. Specifically, they must respond whether they believe that it is true or false that depression is something that only happens to “weak” people; if we are depressed it means that we will always be in that way; if a depressed person cannot be helped; whether people with mental disorders are able to work, especially those diagnosed with schizophrenia, and those who are more dangerous, aggressive and unpredictable. If the users do not respond properly, they are told why their answer is not correct and they will be asked the question again.

*Dali’s Runner*: This minigame is inspired in “runners” games and based on the theme of the painter Salvador Dali. The user has to collect the paintings of this artist while avoiding obstacles in as little time as possible. Once the game is over, a brief biography is shown about the painter, who suffered from a mental illness.

*Stigma Shooter*: This minigame contains concepts related to mental illness which have either a positive or negative connotation. The user has to ‘trap’ the positive concepts (such as overcoming, improving, independence, acceptance, capacity to work) and ‘destroy’ the negative concepts (unpredictable, dangerous, vague, or aggressive) by shooting them.

### Artificial Intelligence

Artificial intelligence was used to control some aspects of the game, namely, background character movement (Merrick and Maher, 2009; Lara-Cabrera et al., 2015) and vehicle movement (Chan et al., 2015). Background character movement is based on *waypoints* (White, 2007), which are a series of 3D coordinates that pinpoint the location of an element in an environment. On the maps, there are a series of interconnected waypoints which represent the path that each of the characters follows. There are various defined paths in each scenario in order to provide a greater sense of realism. The maps are not totally flat, so the height of the nodes was adjusted for any change in height and each node was programmed so the character had a different walking speed and did not appear to move like a robot.

**FIGURE 4:**
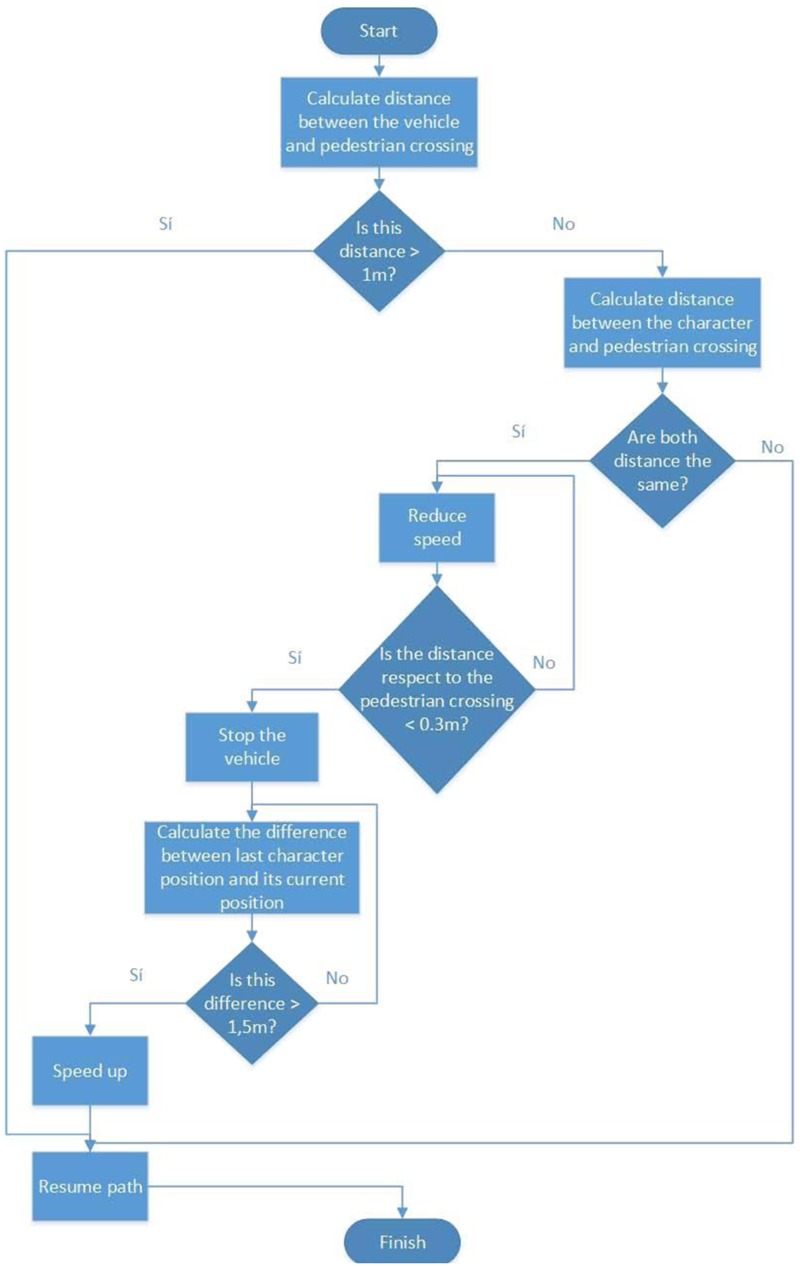
Flow diagram for artificial intelligence of vehicles.

The protagonist can move freely throughout the environment, but the streets have certain restrictions. They cannot be crossed at simply any point, but instead must be crossed at pedestrian crossings. This restriction conditions the movement of the cars in the game, bearing in mind that an algorithm had to be programmed to detect when a character was located at a pedestrian crossing and reduce car speed until coming to a stop. Once it detects that the character has crossed, the vehicle continues driving, as can be seen in **Figure 4**.

### Database Connection

The system communicates with a MySQL database to store relevant game information. Said information is comprised of data related to the user registration and that which the study seeks to obtain from the video game itself. All the data are gathered from the surveys included in the game, user actions, and minigame scores.

**FIGURE 5:**
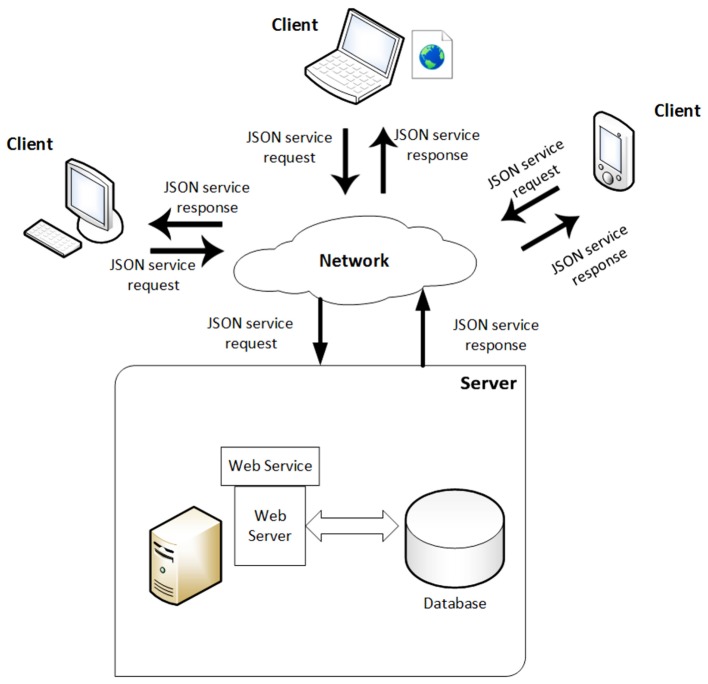
Web services architecture.

Communication with the database to obtain and store data is carried out by means of web services (see **Figure 5**) implemented in PHP with an HTTP Apache server. The technology utilized is REST (Pautasso, 2014), with data encapsulated in JSON (Thu and Aung, 2015). The choice was made to utilize REST as it allows quick access to each physical object with only one request, meaning less workload for the server and better navigation for the user. Furthermore, the system is highly scalable with REST as the servers do not save information of any of the clients and this technology allows cache storing of certain information by making use of corresponding HTTP headers. This last characteristic reduces the workload of the server considerably and improves response time.

### Procedure

The experimental activities were carried out at the University of Almería during school trips to the campus by various secondary schools in the province to learn about the university. The study was carried out over the course of 3 months in sessions held once a week in which two groups of students participated every hour. This study was carried out in accordance with the recommendations of American Psychology Association. The entire experiment was conducted in accordance with the Declaration of Helsinki. All participants gave also oral informed consent. Ethics approval was obtained from the Research Ethics Committee of the University of Almería, Spain.

Tests were conducted in a sound-proofed room equipped with tables, chairs, and a computer connected to a projector. The groups consisted of between 20 and 30 students, and the experiment lasted approximately 1 h. Participation was voluntarily. If any student did not wish to take part, he orshe could stop the activity at any time. The only criterion for exclusion was if the student did not understand Spanish. The students were informed of these details prior to starting the experiment. None of the students were excluded because of the language criterion, nor did anyone choose to leave during the sessions.

The students answered the *Questionnaire on student attitudes toward schizophrenia* both before contact with Stigma-Stop and after. In addition, once the test had ended, they were also asked to evaluate the usefulness and visual appeal of the program (on a scale from 0 to 10), whether they would recommend playing the game to a friend or not, what they thought the game had taught them, and what improvements they would make on the game. In the control group, the students participated in another video game not related to mental health with the same duration as Stigma-Stop. This group also answered the abovementioned questionnaire both before and after playing.

### Statistical Analysis

To analyze the existence of statistically significant differences in the pre-test and post-tests measurements of the two groups, the Student’s *t* was utilized for independent samples. This was complemented by an effect size with a statistic that corresponded, which in this case was Cohen’s *d*. In a second analysis, the post-test and pre-test measurements of each group were compared using the Student’s *t* for related samples. The third analysis involved the use of Cohen’s *d* to assess the magnitude of the change produced during the experience. Finally, the descriptive statistics were utilized which were gathered from the participants’ evaluations and answers. The analysis was carried out using the statistics program SPSS 23.0.

As can be observed in **Table 1**, the average difference test between the pre-test measurements of the experimental group and the control group did not reveal the existence of statistically significant differences between the two with the variables analyzed. However, there were statistically significant differences between the two groups for all of the variables evaluated following the intervention. By using Cohen’s *d*, it was confirmed that the differences between the groups after the activity were moderate.

**Table 1:** Student’s *t*-test for independent samples of pre-test and post-test differences between the experimental group and control group.

	Pre-test			Post-test		
	*t*	*p*	*d*	*t*	*p*	*d*
Dangerousness	0.833	0.405	0.127	-3.477	0.001	-0.481
Stereotypes	-1.146	0.252	-0.280	-3.815	0.000	-0.533
Total	-1.069	0.286	-0.165	-3.894	0.000	-0.597

The means and standard deviations of the variables in the study that correspond to the experimental and control groups for each study phase are displayed in **Table 2**.

**Table 2:** Means and standard deviations of pre-test, post-test and Student’s *t*-test for related samples of post-test–pre-test differences in the study variables in the experimental group and control group.

	Experimental					Control				
	Pre-test	Post-test	Pre–post			Pre-test	Post-test	Pre–post		
	*M (SD)*	*M (SD)*	*t*	*p*	*d*	*M (SD)*	*M (SD)*	*t*	*p*	*d*
Dangerousness	5.11 (2.13)	3.55 (2.56)	16.899	0.000	0.662	4.86 (1.79)	4.66 (2.02)	1.244	0.220	0.104
Stereotypes	4.41 (3.29)	3.96 (3.33)	3.301	0.001	0.136	5.48 (4.27)	6.00 (4.27)	-1.748	0.088	-0.121
Total	9.54 (4.78)	7.54 (5.24)	10.406	0.000	0.399	10.35 (5.03)	10.64(5.15)	-0.768	0.447	-0.056

The analysis of the post-test–pre-test scores of the control group revealed no statistically significant differences with respect to any of the variables evaluated, as can be observed in **Table 2**. However, significant differences were found in the same analysis for the scores of the experimental group, both in the total of the questionnaire score in relation to stigma and its two factors, namely, dangerousness and stereotypes. Consequently, it can be seen how the stigma levels decreased among the people who participated in the Stigma-Stop program. As regards the scope of the effect, it was observed that the program had a strong impact on reducing stigma in dangerousness but wasweak in terms of affecting other stereotypes.

Regarding the assessment carried out by the participants, the program was given a high score for usefulness (7.8 average) and a slightly lower average score for interest (6.3). 75% of the participants said they would recommend playing Stigma-Stop to a friend.

With regards to the characteristics of the different characters in the game, it can be observed that participants easily identified that said characters were not emotionally well (96.8% for panic disorder with agoraphobia, 96.1% for depression and 86.8% for schizophrenia). However, in the case of bipolar disorder, the percentage was lower (61.8%). The most easily identifiable disorders are precisely those which are most likely to provoke acts of help (90 and 82.4% for depression and panic disorder with agoraphobia, respectively). In contrast, in the case of bipolar disorder and schizophrenia the percentage is reduced to just over half (53 and 62.5% respectively). The character disorder which the participants most identified with (Question 3: Have you ever felt like this character?) was the case of depression (40%). On the other hand, the symptomatology they least identified with was that of schizophrenia (only 5.5% of the participants declared having felt like this character at some time). Panic disorder with agoraphobia and bipolar disorder registered at approximately 15% (16.2 and 15.9% specifically) (**Table 3**).

**Table 3:** Participants’ answers about characters.

	Panic disorder with agoraphobia		Schizophrenia		Bipolar disorder		Depression	
	YES	NO	YES	NO	YES	NO	YES	NO
*Do you think the character is emotionally well?*	3.2%	96.8%	13.2%	86.8%	38.2%	61.8%	3.9%	96.1%
*Do you think you could help this person?*	82.4%	16.4%	62.5%	37.5%	53%	47%	90.0%	10.0%
*Have you ever felt like this character?*	16.2%	83.8%	5.5%	94.5%	15.9%	84.1%	40%	60%

## Discussion

The results of the application of Stigma-Stop with secondary school students are presented. Firstly, it is worth highlighting that with regard to stigma, evaluated using the *Questionnaire on student attitudes toward schizophrenia*, there was a considerable decrease among the participants who used the serious game. This was not the case of the participants who used other video games not related to mental health. As a result, the game’s effectiveness in reducing misconceptions is clearly observed, particularly in relation to dangerousness, in addition to its capacity to increase willingness to approach individuals with mental disorders.

It must be taken into account that the preconceived notion of violence or danger associated with people with severe mental disorders, such as schizophrenia, is the most widespread notion among the general population (Walsh et al., 2002; Angermeyer and Dietrich, 2006). The information and dynamics of Stigma-Stop have been shown to be effective in changing this idea. The same belief that is dispelled the fastest by other anti-stigma programs that provide information on these mental disorders (Bono, 2012). Additionally, students who either know someone with a mental illness or already have knowledge on the subject recognize that these individuals are not as dangerous as people would think. Consequently, even though we are dealing with a rather common misconception, it seems that its effects can be mitigated by supplying adequate information.

However, in order to reduce other stereotypes, it might be necessary to implement other actions, such as establishing direct contact with people suffering from mental disorders, thereby giving students the opportunity to personally meet and get to know these individuals (Pinfold et al., 2005). It is not only a matter of providing information; it is also a question of creating opportunities to interact with people with mental health problems so that the benefit of the intervention can be as effective as possible. In this way, Stigma-Stop could be quite useful for reaching students because it uses an appealing and familiar format in order to address issues of mental health. Nevertheless, direct contact with afflicted individuals would be additionally benefitial. In fact, it has been demonstrated that contact with people that have mental disorders is the most important strategy for reducing levels of stigma (Stuart and Laraia, 2006; Clement et al., 2013). It could be concluded that Stigma-Stop and direct interaction are completely compatible and suitable methods for these purposes. This aspect will most certainly be the focus of future research.

It is important to note that the young people who participated in the study correctly identified that all the characters were not psychologically well. The only one that raised any doubt corresponded to bipolar disorder, which some students likely consider to be more a characteristic of “ego” than a psychological alteration. It is therefore necessary that future research investigate as to whether young people simply do not recognize bipolar disorder as an illness as easily (Smith et al., 2011), or if the character itself in Stigma-Stop, at least for young people, does not clearly display the key traits to understand that it is a disorder.

Similarly, most students responded that they would be able to help individuals with disorders regarded as common (such as depression and panic disorder with agoraphobia) while on the contrary their responses were lower for bipolar disorder and schizophrenia. Finally, 40% of the participants recognized they had once felt like the character with depression. This percentage was lower with regards to bipolar disorder and panic disorder. The lowest percentage of participants who thought they had at one time experienced the same feelings as the characters was in the case of schizophrenia. This coincides with epidemiological studies concerning the frequency of these disorders (Kessler et al., 2012).

The general assessment of Stigma-Stop by the students revealed interesting results as well. The participants scored close to eight points (7.8) for the game’s usefulness and a slightly lower score for interest (average score of 6.3). Some of the students’ comments made about the game were the following: “everyday examples help to understand these disorders a little better,” “it is fundamental to help and accept any individual, and to be patient,” “people with schizophrenia as not necessarily aggressive,” and “I have learnt that I should treat people with these problems naturally.” Additionally, 75% stated they would recommend trying this game to a friend.

In light of the results obtained, Stigma-Stop has proved to be an effective tool for raising awareness among young people about mental health problems and for providing information about these disorders, which makes it possible to dispel myths and misconceptions. It constitutes a dynamic and fun method of presenting the issue and being able to discuss it later on in class.

As regards limitations, the present study failed to evaluate the mental state of the participating students (whether or not they themselves had a mental disorder), an aspect that could also influence the results, and no follow-up was made to assess whether changes to stigma among participants were maintained. Neither did the study address other sociodemographic characteristics such as the religion, socioeconomic status, family history of mental health or knowledge about mental disorder of the participants, which may have also influenced the results. These constitute important factors and variables that will be provided for in future studies. Similarly, the present study evaluated stigma with a tool focusing on schizophrenia. Although the highest levels of stigma are associated with said disorder (Crisp, 2001; Angermeyer and Dietrich, 2006; López et al., 2008), it is also important that future research continue to evaluate the effect of the other specific disorders addressed herein (i.e., as bipolar disorder, depression and agoraphobia).

## Author Contributions

AC: contribution to the conception, coordination, and design of the work; NN and JG: contribution to the implementation of the program and introduction of the data in the database. JMAP: contribution to data analysis and methodological aspects. JO, DC, and JAP: contribution in the computer programming of the video game.

## Conflict of Interest Statement

The authors declare that the research was conducted in the absence of any commercial or financial relationships that could be construed as a potential conflict of interest.
